# Assessing the impact of Spirulina supplementation on the growth of children and adolescents: a systematic review and meta-analysis

**DOI:** 10.3389/fnut.2026.1779491

**Published:** 2026-03-16

**Authors:** Bijaya Kumar Mishra, Jaya Singh Kshatri, Bharati Kulkarni, Swagatika Pati, Harshita Dhusiya, Pritimayee Sethy, Tanveer Rehman, Aparna Mukherjee, Srikanta Kanungo, Sanghamitra Pati

**Affiliations:** 1 ICMR–Regional Medical Research Centre, Bhubaneswar, India; 2 ICMR-National Institute of Nutrition, Hyderabad, India; 3 Indian Council of Medical Research, New Delhi, India

**Keywords:** children and adolescents, growth, nutrient, nutritional supplementation, Spirulina

## Abstract

**Background:**

Spirulina, a nutrient-dense blue-green microalgae, has been proposed as a sustainable intervention to combat undernutrition in children and adolescents. Despite its nutritional benefits, evidence regarding its impact on overall growth in this population remains limited and inconsistent. This systematic review and meta-analysis synthesizes the available evidence on impact of Spirulina supplementation on the growth of children and adolescents.

**Methods:**

Following PRISMA (version 2020) guidelines, we systematically searched five databases [PubMed, Embase, CINAHL, CENTRAL and Google Scholar (till 16^th^ July 2024)] for experimental studies published in English. Eligible studies assessed the impact of Spirulina supplementation on the growth of children and adolescents (<18 years), with growth-related outcomes such as changes in height, weight, etc. Data extraction and risk of bias assessment were conducted independently. A random-effects meta-analysis was performed using standardized mean differences (SMDs) to pool results.

**Findings:**

Of 208 identified studies, 5 met the inclusion criteria, and 2 were included in the meta-analysis. The pooled SMD for weight changes was −0.526 (95% CI, −1.289 to 0.236), indicating no statistically significant effect (*p* = 0.176). Heterogeneity was substantial (I^2^ = 99%). Variability in intervention dosage, duration and adherence to supplementation contributed to the observed heterogeneity.

**Interpretation:**

Spirulina supplementation did not show a statistically significant impact on growth outcomes in children and adolescents. Further high-quality studies are needed to explain its role as a nutritional intervention.

**Systematic review registration:**

https://www.crd.york.ac.uk/PROSPERO, identifier: CRD4202457183.

## Introduction

Adequate nutrition is vital for children and adolescents as it helps them to have proper growth and development by laying the foundation of their health and well-being (1). Optimal nutrition provides the essential building blocks for healthy growth and a strong immune system in children (2, 3). Nutritional deficiencies during childhood can lead to serious health issues, including undernutrition, weakened immune function, susceptibility to infectious diseases, cognitive impairments, low intellectual quotient and behavioral problems (4–8). Inadequate intake of nutrition leads to compromised nutritional status, characterized by weight and/or height that is lower than the standards for an individual’s age (9, 10).

Undernutrition continues to be a major global public health problem among children and adolescents, particularly in low-and middle income countries (LMICs), where growth faltering remains highly prevalent. Growth failure during childhood and adolescence reflects prolonged deficiencies in energy, protein, and essential micronutrients and is associated with increased morbidity, impaired physical and cognitive development, and adverse health outcomes later in life (8).

A less expensive and practical approach to solve this problem is to use locally obtainable, multi-micronutrients dense foods that can complement regular household diets and support growth during critical developmental periods (11). Algae have been suggested as a source of protein (12). *Arthrospira platensis* or Spirulina is a blue-green micro-algae (11). It is rich in protein (between 60 and 70%), calcium, phosphorus, iron, gamma-linolenic, linoleic acid, oleic acids, B vitamins, vitamin E and beta carotene (13, 14).

Since ancient times, people have utilized Spirulina for its nutritional benefits (15). Nowadays, Spirulina is evolving as a solution to many other health problems (15, 16). Spirulina is typically consumed in small quantities (0.5–3 g per serving) as a dietary supplement and has been recognized for its safety and nutritional value (17). The Food and Drug Administration (FDA) has approved Spirulina as a dietary supplement and granted it the GRAS certification (Generally Recognized as Safe) (3, 18, 19). As per the Food Safety and Standards Authority of India, Spirulina from Arthrospira platensis is a nutraceutical (20). In the context of child and adolescent nutrition, the relevance of Spirulina lies primarily in its nutrient density and potential to support physical growth through improved protein and micronutrient intake, rather than its broader therapeutic or pharmacological effects (15, 21, 22). Although Spirulina has been investigated for various therapeutic effects, its relevance to child and adolescent growth is mainly attributed to its rich nutritional composition and potential to improve lipid metabolism and overall nutritional status, rather than its broader disease related benefits (15, 23, 24).

Various studies have assessed its health benefits in children. Li. et al. found it to raise vitamin A stores in children (25). Another study found that a particular combination of parboiled rice, cashew and Spirulina was an effective weaning food for children between 6 to 24 months of age (26). Several experimental studies remain variables and context-specific. Difference in study populations, and outcome assessment have resulted in mixed and sometimes inconclusive evidence regarding its effectiveness in improving child and adolescent growth.

Although individual experimental studies are available, the existing evidence has not been systematically synthesized to clarify these inconsistencies or to quantify the overall effect of Spirulina supplementation on growth outcomes in children adolescents. Therefore, the absence of consolidated evidence, combined with variability in existing findings, provides a strong rationale for undertaking a systematic review and meta-analysis to critically appraise the available literature and generate clearer, policy-relevant evidence. Hence, in our study, we endeavored to assess the same.

## Objective

To assess the impact of Spirulina supplementation on the growth of children and adolescents (<18 years of age).

## Methodology

We did this study following the PRISMA guidelines (27, 28). We registered the protocol of the study in PROSPERO with ID CRD42024571836.

### Search

Using the MeSH search terms “Spirulina” and “Pediatric” and “Adolescents,” we did a thorough and systematic literature search for studies published in the English language, in databases like PubMed, CENTRAL, CINAHL, Embase and Google Scholar. The literature search was from database inception till 15th july 2024.

### Search strategy

The search strategy combined controlled vocabulary (MeSH terms) and free-text keywords related to Spirulina supplementation and the pediatric population. Boolean operators (AND, OR) were used to combine search terms. The following search strings were applied:

(“Spirulina”[MeSH Terms] OR “Spirulina”[Title/Abstract] OR “Arthrospira maxima”[Title/Abstract] OR “Spirulina maxima”[Title/Abstract] OR “Spirulina platensis”[Title/Abstract] OR “Arthrospira platensis”[Title/Abstract])

AND

(“Paediatric”[MeSH Terms] OR “Paediatric”[Title/Abstract] OR “Young adults”[Title/Abstract] OR “Adolescents”[Title/Abstract] OR “Children*”[Title/Abstract]).

Titles and abstracts were reviewed by three independent reviewers in Covidence (JSK, SP, HRD).

### Study selection

As per the predefined eligibility criteria for inclusion and exclusion of studies, the title and the abstract of the identified studies were screened by reviewers (JSK, SP, HRD). Full-text screening of potentially relevant studies was carried out by reviewers (JSK, TR, SP, HRD, PS). The whole process of selection of studies was done independently and in duplicate in Covidence software.

### Criteria for inclusion/exclusion of studies

Study design: Only experimental studies reporting on Spirulina intervention and its impact on the growth of children and adolescents were included irrespective of settings. We considered only those studies which have been published in English in peer-reviewed journals. Observational studies, studies not reporting primary data (e.g., commentaries, editorials), studies with inadequate information in the title/abstract, dissertations and conference proceedings were excluded. Studies focusing solely on clinical outcomes without assessing compliance with Spirulina supplementation and studies involving participants with specific disease conditions were excluded. Although there is a considerable amount of grey literature on this topic, we limited our search to peer-reviewed articles to ensure the inclusion of studies.

Population/Participants: Studies involving children and adolescents aged less than 18 years at the time of the intervention were included.

Intervention(s)/exposure(s)/phenomenon of interest: The intervention of interest was Spirulina supplementation, either as a standalone supplement or as part of a mixed intervention, provided in any form or dosage.

Comparator/context: Studies with any control group that did not receive Spirulina supplementation or received a placebo were included.

Outcome: The included articles were reviewed for the following outcomes: Impact of Spirulina on the growth of children and adolescents, considering factors like change in height and weight.

### Risk of bias assessment

The quality of the methods used in the included studies was appraised with the help of the Cochrane Risk of Bias Tool version 2.0 (29, 30). For each domain, it was leveled as either high, low, or some concerns. Again, the studies were evaluated for their overall risk of bias, which was determined to be either low, some concerns, or high (30). Detailed domain-wise and overall risk of bias judgments for all included studies are presented in Supplementary Table 1.

### Data extraction, synthesis, and analysis

**Figure 1:**
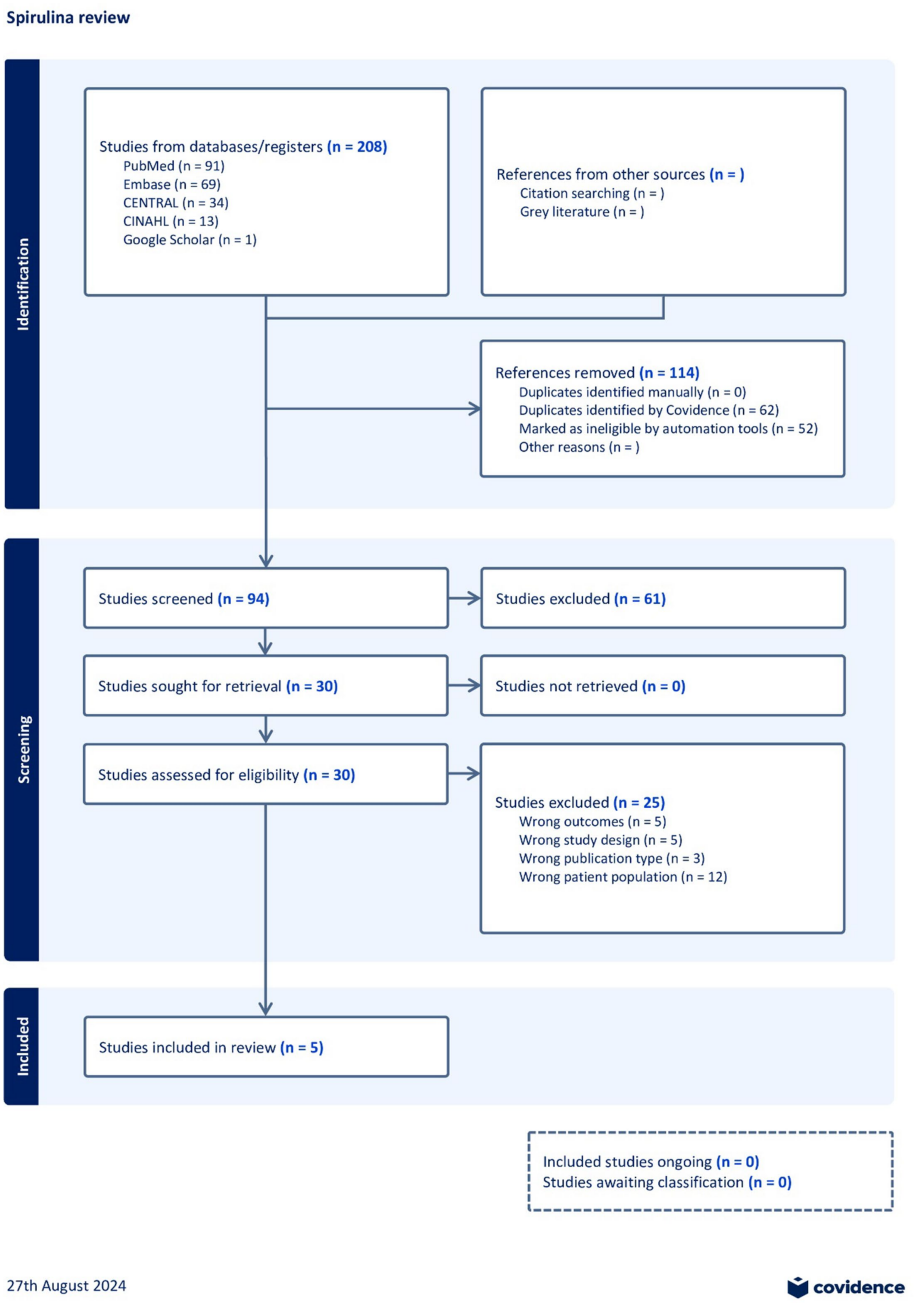
PRISMA flow diagram.

Data extraction was done by 3 independent reviewers manually in Covidence (SP, HRD, PS) and conflicts were resolved in consultation with a 4th reviewer (TR). Data extraction was done for study aspects like title, year, type, author, design, setting, country, sample size, participants, interventional and control group with baseline and endline. For the data synthesis, we employed a random-effects model to account for variability between studies. Standardized mean differences (SMDs) were calculated with 95% confidence intervals for continuous outcomes. The primary outcome measure was the effect size for the intervention versus control groups, reported as weighted means. All analyses were conducted using Stata 14 software. Forest plot was generated to visualize the effect sizes of individual studies and the overall pooled estimate. The weight assigned to each study were determined by the random-effect model, allowing for variations in sample size and study precision (Figure 1).

## Results

By utilizing the Medical Subject Heading (MeSH) terms (31) and keywords “Spirulina,” “Pediatric,” and “Adolescents,” we conducted a search that resulted in 91 studies from PubMed, 69 from Embase, 34 from CENTRAL, 13 from CINAHL, and 1 from Google Scholar using the query “Spirulina and Pediatric and Adolescents”. Thus, the search yielded 208 studies. Out of the initial 208 studies, Covidence identified 62 duplicates, and 52 references were marked as ineligible by automation tools. Hence a total of 114 studies were excluded from the 208 studies. 94 studies were assessed for title and abstract screening, out of which 30 studies were chosen for full-text screening.

After full text screening, we excluded 25 studies from the review for various reasons as follows. 5 studies were excluded for reporting different outcomes, while 5 were excluded for using wrong study design. 3 studies were removed because they were not published in the required format, e.g., commentaries or editorials. Finally, 12 studies were excluded because the study population did not match the criteria defined for the systematic review, i.e., the study population were not children below18 years of age.

Ultimately, 5 studies, which fulfilled the eligibility criteria, were selected for extraction of data (10, 17, 32–34). The PRISMA statement and flowchart, which detail the methods of data extraction and abstraction, are included at the end of the manuscript.

Finally, 2 studies were included in the meta-analysis. The 2 studies were *Masuda et al., 2019* and *N et al., 2016*, both of which provided data on change in body weight as an outcome (10, 34).

The five included studies were experimental in nature and evaluated the effects of Spirulina supplementation on growth-related outcomes among children and adolescents. Study populations varied in terms of age groups, baseline nutritional status, and geographic settings. The duration of Spirulina supplementation and dosage differed across studies, contributing to heterogeneity in intervention protocols.

The outcomes assessed across studies included anthropometric indicators such as body weight, height, and growth parameters, though not all studies reported comparable outcomes or sufficient data for quantitative pooling. Due to variations in outcome reporting and study design, a narrative synthesis was undertaken for all included studies, while meta-analysis was conducted only for outcomes with sufficient comparable data.

Across the included studies, the findings regarding the impact of Spirulina supplementation on growth outcomes were mixed. Some studies reported improvements in anthropometric measures, particularly among children with compromised nutritional status at baseline, while others found minimal or no statistically significant differences between intervention and control groups. Differences in supplementation duration, dosage, baseline nutritional status, and outcome measurement methods likely contributed to the variability in findings.

Studies that assessed outcomes other than body weight suggested potential benefits of Spirulina on nutritional recovery and growth trends, though the evidence was inconsistent and limited by small sample sizes and short follow-up periods. Overall, while Spirulina supplementation appeared to be well tolerated, the strength of evidence supporting its effectiveness on growth-related outcomes in pediatric and adolescent populations remains limited.

### Meta-analysis results

Random-effects inverse-variance model was used to pool the aggregate data from the studies. The overall effect size was calculated as a standardized mean difference (SMD).

- Masuda et al. showed a small negative effect size of −0.141 [95% CI, −0.151, −0.132], contributing 50.50% of the weight to the meta-analysis.
- Abed et al. showed a much larger negative effect size of −0.919 [95% CI, −1.072, −0.767], contributing 49.50% of the weight.

**Figure 2:**
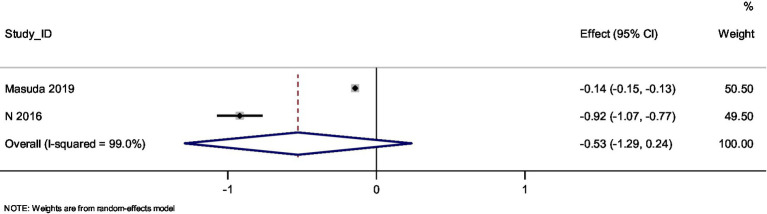
Forest plot.

The overall pooled effect size across both studies was −0.526 [95% CI, −1.289, 0.236], suggesting no statistically significant impact of Spirulina supplementation on change in body weight in the included population (*p* = 0.176; Figure 2; Table 1).

**Table 1:** Summary of included Spirulina supplementation studies and reported outcomes.

Sr.no	Article ID	Age group	Spirulina dose	Duration	Growth outcomes
1	Masuda et al. 2019 (10, 11)	Infants, aged between (6 and 18 months)	Not mentioned	12 months	children’s gross motor and fine motor skills, Mental development
2	Barennes et al. 2022 (17)	Children (4–7 years)	2 gms per day	10 months	Increase in weight and height
3	Othoo et al. 2021 (32)	children aged (6–23 months)	0.4% Spirulina powder in Spirulina Corn Soy Blend flour thrice a day	6 months	recovery from iron deficiency
4	Simpore et al. 2006 (33)	children aged <5 years	5 gms of Spirulina twice a day	2 months	increment of weight,
5	Abed et al. 2016 (34)	children aged <5 years	3 gms of Spirulina.	3 months	Increase in weight and height, improvement in the level of serum iron and increase in ferritin levels

### Heterogeneity

Significant heterogeneity was detected across the studies (Cochran’s Q = 99.87, *p* < 0.001), with an I^2^ value of 99.0%, indicating substantial between-study variability. The tau^2^ value was calculated as 0.2995, reflecting moderate heterogeneity in the effect size estimates.

A very high degree of heterogeneity was observed between the included studies (I^2^ = 99%; Cochran’s Q = 99.87, *p* < 0.001), indicating substantial between-study variability. This extreme level of heterogeneity severely limits the interpretability and generalizability of the pooled effect estimate, and the summary effect should therefore be interpreted with considerable caution. Given that only two studies were eligible for inclusion in the meta-analysis, further exploration of heterogeneity through subgroup analyses or sensitivity analyses was not feasible.

## Discussion

To the best of our knowledge, this is the first systematic review and meta-analysis to assess the impact of Spirulina supplementation on the growth of children and adolescents. Our study suggests that Spirulina supplementation does not produce a statistically significant effect on the growth of children and adolescents, particularly their body weight. The pooled effect size of −0.526 is not large enough to confirm a positive impact on weight gain, and the confidence intervals cross zero, indicating uncertainty regarding the true effect of Spirulina supplementation.

Despite the overall negative findings, there was considerable heterogeneity between the studies, as evidenced by the high I^2^ value. This may be attributed to variations in study populations, intervention duration, Spirulina dosage, and potentially adherence to supplementation. The study by Masuda et al. showed only a small negative effect, while that by Abed et al. reported a much larger impact. The variation in study outcomes might reflect contextual differences in the populations studied, nutritional status at baseline, or the formulation of Spirulina used in the studies.

Our meta-analysis suggested that Spirulina supplementation did not significantly improve the body weight of children and adolescents. Similar to our study, a meta-analysis that included participants of different age groups found that Spirulina had no significant effect on body weight (35). Interestingly enough, some studies have even found Spirulina to be effective in reduction of body weight in obese individuals (36, 37).

The study by Masuda et al. did not find any significant improvement in infant growth indicators in case of Spirulina supplementation in comparison to the control. Nevertheless, the other beneficial effects of Spirulina cannot be ignored. Spirulina has been shown to help in better recovery from iron deficiency among children (32, 38–41).

Though Spirulina does not significantly influence growth in children and adolescents directly, various studies have found it to be a beneficial nutrition supplement in health and disease. It has also been found to be free from toxicity (14). Nevertheless, production of Spirulina needs appropriate resources, and more economically viable and efficient production methods are currently being explored (42–46). Hence, whether it is used as a food supplement or as a therapeutic supplement, Spirulina should be used judiciously and based on scientific evidence. Moreover, emphasis should be given upon appropriate dosage and proper drug delivery methods in order to maximize the therapeutic effect of Spirulina in recommended health conditions (47). Nevertheless, production of Spirulina requires appropriate resources, and more economically viable efficient production methods are are currently being explored (42). Operational and economic feasibility of spirulina-based biorefineries has also been examined (43). Various cultivation strategies such as attached cultivation have been investigated to improve biomass productivity (44). The use of biogas effluent for spirulina cultivation has been explored as a sustainable approach (45). Additionally, mixotrophic cultivation in dairy wastewater has been studied to enhance biomass production and antioxidant capacity (46).

Our review tried to integrate findings from multiple studies, providing evidence-based recommendations for policymakers and practitioners regarding the use of Spirulina in child and adolescent nutrition programs. While our meta-analysis was limited to two studies, the findings align with some of the broader literature suggesting inconsistent effects of Spirulina on growth. Further research is needed, with larger sample sizes and more robust study designs, to better elucidate the role of Spirulina supplementation in improving nutrition, growth and development of children and adolescents.

### Policy implications

Based on the current evidence, Spirulina supplementation cannot be recommended as a population-level intervention for improving growth outcomes in children and adolescents. The available studies are limited in number, heterogeneous in design, and do not demonstrate a statistically significant or consistent benefit on key growth indicators. As such, the present evidence base is insufficient to justify large-scale policy adoption or integration into national child nutrition programs. There is a critical need for well-designed, adequately powered randomized controlled trials with standardized intervention protocols. Future trials should ensure consistency in dosage, formulation, duration, outcome measurement, and adherence assessment, and should be conducted across diverse geographic and socio-economic settings.

### Limitations

This review is limited by the very small number of studies included in the meta-analysis, with pooled estimates based on only two trials, which restricts statistical power and limits the robustness of the findings. Additionally, substantial heterogeneity across studies and variations in intervention protocols and outcome measures constrain the generalizability of the results.

## Conclusion

This systematic review and meta-analysis examined the available evidence on the impact of Spirulina supplementation on the growth of children and adolescents. The findings indicate that Spirulina supplementation has not consistently demonstrated a clear benefit on growth outcomes, such as weight and height, across the included studies. While Spirulina is a nutrient-dense supplement and has been explored as a potential nutritional intervention in undernourished populations, the current evidence remains limited and variable.

Overall, the result suggest that Spirulina supplementation cannot be conclusively recommended as an effective strategy for improving growth in children and adolescents based on the existing evidence. Further well-designed studies with standardized interventions and outcome measures are needed to better understand its role in child and adolescent nutrition.

## Data Availability

The original contributions presented in the study are included in the article/Supplementary material, further inquiries can be directed to the corresponding author.

## References

[1] Hilger J Goerig T Weber P Hoeft B Eggersdorfer M Carvalho N . Micronutrient intake in healthy toddlers: a multinational perspective. Nutrients. (2015) 7:6938–55. doi: 10.3390/nu7085316, 26295254 PMC4555155

[2] Kozioł-Kozakowska A. Adequate nutrition in early childhood. Children. (2023) 10:1155. doi: 10.3390/children10071155, 37508652 PMC10377795

[3] Kashyap GC Sarala R Manjunath U. Impact of *Spirulina* chikki supplementation on nutritional status of children: an intervention study in Tumkur District of Karnataka, India. Front Pediatr. (2022) 10:860789. doi: 10.3389/fped.2022.860789, 35498815 PMC9051330

[4] Matondo FK Takaisi K Nkuadiolandu AB Kazadi Lukusa A Aloni MN. *Spirulina* supplements improved the nutritional status of undernourished children quickly and significantly: experience from Kisantu, the Democratic Republic of the Congo. Int J Pediatr. (2016) 2016:1–5. doi: 10.1155/2016/1296414 PMC5061973 27777589

[5] Bhutta ZA Berkley JA Bandsma RHJ Kerac M Trehan I Briend A. Severe childhood malnutrition. Nat Rev Dis Primers. (2017) 3:17067. doi: 10.1038/nrdp.2017.67, 28933421 PMC7004825

[6] Siva Kiran R R M GM Satyanarayana SV. Spirulina in combating Protein Energy Malnutrition (PEM) and Protein Energy Wasting (PEW) - A review. (2016). Available online at: http://rgdoi.net/10.13140/RG.2.1.3149.0325 (Accessed on 2024 Sep 27)

[7] Liu J Hanlon A Ma C Zhao S Cao S Compher C. Low blood zinc, Iron, and other sociodemographic factors associated with behavior problems in preschoolers. Nutrients. (2014) 6:530–45. doi: 10.3390/nu6020530, 24473235 PMC3942715

[8] Salam RA Das JK Ahmed W Irfan O Sheikh SS Bhutta ZA. Effects of preventive nutrition interventions among adolescents on health and nutritional status in low- and middle-income countries: a systematic review and meta-analysis. Nutrients. (2019) 12:49. doi: 10.3390/nu12010049, 31878019 PMC7019616

[9] Kesari A Noel JY. “Nutritional Assessment” In: StatPearls. Treasure Island (FL): StatPearls Publishing (2024). 35593821

[10] Masuda K Chitundu M. Multiple micronutrient supplementation using *spirulina platensis* and infant growth, morbidity, and motor development: evidence from a randomized trial in Zambia. PLoS One. (2019) 14:e0211693. doi: 10.1371/journal.pone.0211693, 30759117 PMC6373937

[11] Masuda K Chitundu M. Multiple micronutrient supplementation using *Spirulina platensis* during the first 1000 days is positively associated with development in children under five years: a follow up of a randomized trial in Zambia. Nutrients. (2019) 11:730. doi: 10.3390/nu11040730, 30934863 PMC6520735

[12] Espinosa-Ramírez J Mondragón-Portocarrero AC Rodríguez JA Lorenzo JM Santos EM. Algae as a potential source of protein meat alternatives. Front Nutr. (2023) 10:1254300. doi: 10.3389/fnut.2023.1254300, 37743912 PMC10513374

[13] Spirulina" In: Drugs and lactation database (LactMed®). Bethesda (MD): National Institute of Child Health and Human Development (2006)

[14] Chamorro-Cevallos G. ASPECTOS NUTRICIONALES y TOXICOLÓGICOS de SPIRULINA (ARTHROSPIRA). Nutr Hosp. (2015) 1:34–40. doi: 10.3305/nh.2015.32.1.9001 26262693

[15] Candelaria GC Rosa Virginia GR María Angélica MV Yuliana GM José Melesio CL Germán CC. Effect of *Arthrospira* (*Spirulina*) maxima on cadmium-chloride-induced alterations in sexual behavior and fertility in male Wistar rats. Pharmaceuticals. (2024) 17:332. doi: 10.3390/ph17030332, 38543118 PMC10975135

[16] Sinha S Patro N Patro IK. Maternal protein malnutrition: current and future perspectives of *Spirulina* supplementation in neuroprotection. Front Neurosci. (2018) 12:966. doi: 10.3389/fnins.2018.00966, 30618587 PMC6305321

[17] Barennes H Houdart L de Courville C Barennes F. *Spirulina* as a daily nutritional supplement of young pre-school Cambodian children of deprived settings: a single-blinded, placebo-controlled, cross-over trial. BMC Pediatr. (2022) 22:701. doi: 10.1186/s12887-022-03766-5, 36476193 PMC9727933

[18] AlFadhly NKZ Alhelfi N Altemimi AB Verma DK Cacciola F Narayanankutty A. Trends and technological advancements in the possible food applications of *Spirulina* and their health benefits: a review. Molecules. (2022) 27:5584. doi: 10.3390/molecules27175584, 36080350 PMC9458102

[19] Trotta T Porro C Cianciulli A Panaro MA. Beneficial effects of *Spirulina* consumption on brain health. Nutrients. (2022) 14:676. doi: 10.3390/nu14030676, 35277035 PMC8839264

[20] Ahmed N Sheikh MA Ubaid M Chauhan P Kumar K Choudhary S. Comprehensive exploration of marine algae diversity, bioactive compounds, health benefits, regulatory issues, and food and drug applications. Measurement: Food. (2024) 14:100163. doi: 10.1016/j.meafoo.2024.100163

[21] Al-Dhabi NA Valan Arasu M. Quantification of phytochemicals from commercial *Spirulina* products and their antioxidant activities. Jayaprakasha GK, editor. Evid Based Complement Alternat Med. (2016) 2016:7631864. doi: 10.1155/2016/7631864 26933442 PMC4737012

[22] Juszkiewicz A Basta P Petriczko E Machaliński B Trzeciak J Łuczkowska K . An attempt to induce an immunomodulatory effect in rowers with *Spirulina* extract. J Int Soc Sports Nutr. (2018) 15:9. doi: 10.1186/s12970-018-0213-3 29467598 PMC5819236

[23] Masten Rutar J Jagodic Hudobivnik M Nečemer M Vogel Mikuš K Arčon I Ogrinc N. Nutritional quality and safety of the spirulina dietary supplements sold on the Slovenian market. Foods. (2022) 11:849. doi: 10.3390/foods11060849, 35327271 PMC8954120

[24] Nagaoka S Shimizu K Kaneko H Shibayama F Morikawa K Kanamaru Y . A novel protein C-phycocyanin plays a crucial role in the hypocholesterolemic action of *Spirulina platensis* concentrate in rats. J Nutr. (2005) 135:2425–30. doi: 10.1093/jn/135.10.2425, 16177207

[25] Li L Zhao X Wang J Muzhingi T Suter PM Tang G . Spirulina can increase total-body vitamin a stores of Chinese school-age children as determined by a paired isotope dilution technique. J Nutr Sci. (2012) 1:e19. doi: 10.1017/jns.2012.21, 25191548 PMC4153073

[26] Mahouli LM Saah BF Dongmo H Kenfack JO Njapndounke B Ndjang MMN . Nutritional and rheological characterization of an infant flour based on parboiled rice (*Oryza sativa*), spirulina (*Spirulina platensis*), and cashew nut (*Anacardium occidentale*). Int J Food Sci. (2022) 2022:1–9. doi: 10.1155/2022/3784317 PMC9463026 36089942

[27] Moher D Liberati A Tetzlaff J Altman DG The PRISMA Group. Preferred reporting items for systematic reviews and meta-analyses: the PRISMA statement. PLoS Med. (2009) 6:e1000097. doi: 10.1371/journal.pmed.1000097, 19621072 PMC2707599

[28] Page MJ McKenzie JE Bossuyt PM Boutron I Hoffmann TC Mulrow CD . The PRISMA 2020 statement: an updated guideline for reporting systematic reviews. BMJ. (2021) 372:n71. doi: 10.1136/bmj.n71 33782057 PMC8005924

[29] Cumpston M Li T Page MJ Chandler J Welch VA Higgins JP . Updated guidance for trusted systematic reviews: a new edition of the Cochrane handbook for systematic reviews of interventions. Cochrane Database Syst Rev. (2019) 10:2. doi: 10.1002/14651858.ED000142, 31643080 PMC10284251

[30] Sterne JAC Savović J Page MJ Elbers RG Blencowe NS Boutron I . RoB 2: a revised tool for assessing risk of bias in randomised trials. BMJ. (2019) 366:l4898. doi: 10.1136/bmj.l4898, 31462531

[31] Rogers FB. Medical subject headings. Bull Med Libr Assoc. (1963) 51:114–6. 13982385 PMC197951

[32] Othoo DA Ochola S Kuria E Kimiywe J. Impact of Spirulina corn soy blend on Iron deficient children aged 6–23 months in Ndhiwa Sub-County Kenya: a randomized controlled trial. BMC Nutr. (2021) 7:70. doi: 10.1186/s40795-021-00472-w, 34749821 PMC8577024

[33] Simpore J Kabore F Zongo F Dansou D Bere A Pignatelli S . Nutrition rehabilitation of undernourished children utilizing Spiruline and Misola. Nutr J. (2006) 5:3. doi: 10.1186/1475-2891-5-3 16430775 PMC1386687

[34] Abed E Ihab AN Suliman E Mahmoud A. Maternal and pediatric nutrition journal impact of Spirulina on nutritional status, haematological profile and anaemia status in malnourished children in the Gaza strip: randomized clinical trial. Matern Pediatr Nutr J. (2016) 110:2. doi: 10.4172/2472-1182.1000110

[35] Huang H Liao D Pu R Cui Y. Quantifying the effects of spirulina supplementation on plasma lipid and glucose concentrations, body weight, and blood pressure. Diabetes Metab Syndr Obes Targets Ther. (2018) 11:729–42. doi: 10.2147/DMSO.S185672 PMC6241722 30532573

[36] Zeinalian R Farhangi MA Shariat A Saghafi-Asl M. The effects of Spirulina platensis on anthropometric indices, appetite, lipid profile and serum vascular endothelial growth factor (VEGF) in obese individuals: a randomized double blinded placebo controlled trial. BMC Complement Altern Med. (2017) 17:225. doi: 10.1186/s12906-017-1670-y, 28431534 PMC5399840

[37] Hernández-Lepe M López-Díaz J Juárez-Oropeza M Hernández-Torres R Wall-Medrano A Ramos-Jiménez A. Effect of *Arthrospira* (Spirulina) *maxima* supplementation and a systematic physical exercise program on the body composition and cardiorespiratory fitness of overweight or obese subjects: a double-blind, randomized, and crossover controlled trial. Mar Drugs. (2018) 16:364. doi: 10.3390/md16100364, 30275428 PMC6213464

[38] Kaipa VRK Asif SM Assiri KI Saquib SA Arem SA Sree S . Antioxidant effect of spirulina in chronic periodontitis. Medicine (Baltimore). (2022) 101:e31521. doi: 10.1097/MD.0000000000031521, 36550811 PMC9771209

[39] Moradi S Bagheri R Amirian P Zarpoosh M Cheraghloo N Wong A . Effects of Spirulina supplementation in patients with ulcerative colitis: a double-blind, placebo-controlled randomized trial. BMC Complement Med Ther. (2024) 24:109. doi: 10.1186/s12906-024-04400-w, 38424572 PMC10905931

[40] Choi WY Lee WK Kim TH Ryu YK Park A Lee YJ . The effects of Spirulina *maxima* extract on memory improvement in those with mild cognitive impairment: a randomized, double-blind, placebo-controlled clinical trial. Nutrients. (2022) 14:3714. doi: 10.3390/nu14183714, 36145090 PMC9505028

[41] Aghasadeghi MR Zaheri Birgani MA Jamalimoghadamsiyahkali S Hosamirudsari H Moradi A Jafari-Sabet M . Effect of high-dose Spirulina supplementation on hospitalized adults with COVID-19: a randomized controlled trial. Front Immunol. (2024) 15:1332425. doi: 10.3389/fimmu.2024.1332425, 38655258 PMC11036872

[42] AlFadhly NKZ Alhelfi N Altemimi AB Verma DK Cacciola F. Tendencies affecting the growth and cultivation of genus *Spirulina*: an investigative review on current trends. Plants. (2022) 11:3063. doi: 10.3390/plants11223063, 36432792 PMC9693216

[43] Costa JAV Freitas BCB Rosa GM Moraes L Morais MG Mitchell BG. Operational and economic aspects of Spirulina-based biorefinery. Bioresour Technol. (2019) 292:121946. doi: 10.1016/j.biortech.2019.121946, 31422868

[44] Zhang L Chen L Wang J Chen Y Gao X Zhang Z . Attached cultivation for improving the biomass productivity of Spirulina platensis. Bioresour Technol. (2015) 181:136–42. doi: 10.1016/j.biortech.2015.01.025, 25647023

[45] Hultberg M Lind O Birgersson G Asp H. Use of the effluent from biogas production for cultivation of Spirulina. Bioprocess Biosyst Eng. (2017) 40:625–31. doi: 10.1007/s00449-016-1726-2, 28025700 PMC5360822

[46] Pereira MIB Chagas BME Sassi R Medeiros GF Aguiar EM Borba LHF . Mixotrophic cultivation of *Spirulina platensis* in dairy wastewater: effects on the production of biomass, biochemical composition and antioxidant capacity. PLoS One. (2019) 14:e0224294. doi: 10.1371/journal.pone.0224294, 31648264 PMC6812818

[47] ElFar OA Billa N Lim HR Chew KW Cheah WY Munawaroh HSH . Advances in delivery methods of *Arthrospira platensis* (spirulina) for enhanced therapeutic outcomes. Bioengineered. (2022) 13:14681–718. doi: 10.1080/21655979.2022.2100863, 35946342 PMC9373759

