# Supplementary material for: Assessing the impact of Spirulina supplementation on the growth of children and adolescents: a systematic review and meta-analysis
Source: Front Nutr. 2026 Mar 16;13:1779491. doi: 10.3389/fnut.2026.1779491 (PMC13034566; doi:10.3389/fnut.2026.1779491)
Supplement: Supplementary file 1 [file Table_1.docx]

**Supplementary File**

**Table I.** Risk of Bias assessment of included studies using the Cochrane Risk of Bias Tool (RoB 2.0)

| **Study (Year)** | **Randomization process** | **Deviations from intended interventions** | **Missing outcome data** | **Measurement of the outcome** | **Selection of the reported result** | **Overall risk of bias** |
| --- | --- | --- | --- | --- | --- | --- |
| Othoo et al., 2021 | Low risk | Low risk | Low risk | Low risk | Some concerns | Some concerns |
| N et al., 2016 | Some concerns | Some concerns | Low risk | Low risk | Low risk | Some concerns |
| Masuda et al., 2019 | Low risk | Some concerns | Low risk | High risk | Low risk | High risk |
| Simpore et al., 2006 | Low risk | Some concerns | Low risk | High risk | Some concerns | High risk |
| Barennes et al., 2022 | Low risk | Low risk | Low risk | Low risk | Low risk | Low risk |
